# Predicting the Onset of Diabetes with Machine Learning Methods

**DOI:** 10.3390/jpm13030406

**Published:** 2023-02-24

**Authors:** Chun-Yang Chou, Ding-Yang Hsu, Chun-Hung Chou

**Affiliations:** 1 Research Center for Healthcare Industry Innovation, National Taipei University of Nursing and Health Sciences, Taipei 112, Taiwan; 2 Department of Industrial Design, Ming Chi University of Technology, Taipei 243, Taiwan; 3 Industrial Technology Research Institute, Hsinchu 310401, Taiwan

**Keywords:** artificial neural network, supervised learning, confusion matrix, receiver operator characteristic, area under the curve, machine learning, recall, F1 score, deep learning

## Abstract

The number of people suffering from diabetes in Taiwan has continued to rise in recent years. According to the statistics of the International Diabetes Federation, about 537 million people worldwide (10.5% of the global population) suffer from diabetes, and it is estimated that 643 million people will develop the condition (11.3% of the total population) by 2030. If this trend continues, the number will jump to 783 million (12.2%) by 2045. At present, the number of people with diabetes in Taiwan has reached 2.18 million, with an average of one in ten people suffering from the disease. In addition, according to the Bureau of National Health Insurance in Taiwan, the prevalence rate of diabetes among adults in Taiwan has reached 5% and is increasing each year. Diabetes can cause acute and chronic complications that can be fatal. Meanwhile, chronic complications can result in a variety of disabilities or organ decline. If holistic treatments and preventions are not provided to diabetic patients, it will lead to the consumption of more medical resources and a rapid decline in the quality of life of society as a whole. In this study, based on the outpatient examination data of a Taipei Municipal medical center, 15,000 women aged between 20 and 80 were selected as the subjects. These women were patients who had gone to the medical center during 2018–2020 and 2021–2022 with or without the diagnosis of diabetes. This study investigated eight different characteristics of the subjects, including the number of pregnancies, plasma glucose level, diastolic blood pressure, sebum thickness, insulin level, body mass index, diabetes pedigree function, and age. After sorting out the complete data of the patients, this study used Microsoft Machine Learning Studio to train the models of various kinds of neural networks, and the prediction results were used to compare the predictive ability of the various parameters for diabetes. Finally, this study found that after comparing the models using two-class logistic regression as well as the two-class neural network, two-class decision jungle, or two-class boosted decision tree for prediction, the best model was the two-class boosted decision tree, as its area under the curve could reach a score of 0.991, which was better than other models.

## 1. Introduction

Diabetes has become a serious chronic disease in Taiwan in recent years due to changes in demographic structures, westernized diets, and lifestyle changes. As per the International Diabetes Federation (IDF), 6.7 million people worldwide die of diabetes or its complications every year. One person dies of diabetes every five seconds, and the number is continuing to increase [1]. According to the data from Taiwan’s Bureau of National Health Insurance, the prevalence rate of diabetes among adults in Taiwan has reached 5% and is increasing year by year.

The typical symptoms of diabetes are excessive thirst, polyuria, and unexplained weight loss [2]. However, most diabetic patients have no symptoms in the early stage of diabetes. During this period, the diagnosis of diabetes can only rely on blood sampling and follow-up examinations, and there are no other methods to diagnose diabetes according to clinical symptoms [3]. However, such asymptomatic hyperglycemia can cause chronic complications of diabetes, such as eye disease [4], kidney disease, autonomic neuropathy, heart disease, and vascular disease [5]. The regulation of the heart rhythm by the autonomic nerve is a risk factor for cardiovascular events, and is also associated with an increasing risk of total mortality, especially for diabetic patients. The regulation of autonomic nerves to the heart can be detected by means of external stimuli, mechanical manipulation, and drug methods. Heart rate variability (HRV) is the best tool for diagnosing autonomic neuropathy in diabetic patients. In the past, the diagnosis of autonomic nervous system disorders could not be controlled correctly in medical science. Mostly the diagnosis relied on the doctors’ judgment and evaluation scales, but such scales lack scientific data. At present, diagnosis can be achieved through technological instrument detection, such as heart rate variance (HRV), galvanic skin response (GSR), exhaled carbon dioxide concentration (CO_2_) during breathing, measuring different posture blood pressures, and so on [6]. Physicians can judge from the results whether the tester’s autonomic nerves are balanced or if their autonomic nerves are in a hypersympathetic state. Doctors can treat the latter with a combination of symptomatic drugs and psychological treatment. The American Diabetes Medical Association recommends that for patients who are obese, older, have a strong family history of diabetes, or have metabolic syndrome-related factors (such as high triglyceride, low high-density cholesterol, poor plasma glucose tolerance, or high fasting plasma glucose), clinicians should check their fasting plasma glucose to screen for diabetes, so as to improve the diagnosis rate. But even so, the treatment is often delayed for more than half of the people with diabetes because the patients have no symptoms [7].

Therefore, this study used easily obtained information from a hospital’s information system to establish a prediction model for diabetes, and used neural networks to train the model and test the effectiveness of the model. The resulting comparison of various parameters with the predicting results obtained the occurrence of diabetes.

With the right model, doctors should be able accurately diagnose diabetes in future patients, even if they asymptomatic, using eight characteristics: the number of pregnancies, plasma glucose level, diastolic blood pressure, sebum thickness, insulin level, body mass index, diabetes pedigree function, and age [8].

## 2. Literature Review

Diabetes is a collective term for a group of symptoms related to hyperglycemia. It is a chronic metabolic disorder in which patients may have problems with insufficient insulin secretion, insulin resistance, or both. The main clinical symptoms of diabetes are polyuria, thirst, hunger, fatigue, blurred vision, weight loss, and difficulty in wound healing. The American Diabetes Association classifies diabetes into two types. Type I diabetes, formerly known as insulin dependent diabetes mellitus (IDDM), often occurs in childhood, mainly because islet cells are damaged by immune responses. It may be due to the patient’s heredity, living environment, or a viral infection that triggers an autoimmune response that damages the beta cells [9] in the pancreas so that the patient’s body cannot produce enough insulin [10]. Type II diabetes, formerly known as noninsulin dependent diabetes mellitus (NIDDM), is commonly seen in adults and generally occurs when people are about 40 years old. Patients with NIDDM usually have insufficient insulin secretion and insulin resistance concurrently. The cause of Type II diabetes is multifactorial [11,12]. It is generally considered to be related to heredity, obesity, and lack of exercise. Other special types of diabetes include diabetes caused by genetic defects, diabetes caused by pancreatic exocrine destruction, and diabetes caused by drugs or chemicals. Gestational diabetes mellitus (GDM) refers to insulin resistance that is caused by hormonal or metabolic changes during pregnancy [13] (for example, the increase of blood sugar caused by the insufficient compensatory secretion of insulin [14]. This means that diabetes is a disease in which the body does not use blood sugar (glucose) well. Digestion results in food turning it into sugar, which is then turned into energy, and our body produces insulin (a hormone secreted by the pancreas) that diverts excess blood sugar for use by the body’s cells. If you do not get enough insulin, your body can not use the excess sugar, resulting in high blood sugar levels.

The relationship between diabetes and pregnancy is divided into two types: pregestational diabetes and gestational diabetes (GDM). Pregestational diabetes refers to diabetes before pregnancy, which can be divided into insulin-dependent diabetes mellitus (IDDM) (type I) and noninsulin-dependent diabetes mellitus (NIDDM) (type II). For diabetic women, it is best to receive a prenatal consultation before pregnancy, and strictly control blood sugar before pregnancy to ensure that the mother’s body reaches an ideal environment for pregnancy, to reduce the rate of miscarriage. Fetal congenital malformations and mergers symptoms occurs after pregnancy. Insulin injections should be continued or switched to control blood sugar, and oral hypoglycemic drugs should be stopped. Gestational diabetes is diabetes that develops after pregnancy. Such patients account for the majority of pregnant mothers with diabetes. During pregnancy, the metabolism of hormones or carbohydrates will change. As the number of weeks of pregnancy increases, the secretion concentration of hormones, such as human placental prolactin (HPL), estrogen, and progesterone, will increase also, as will the cells’ resistance to insulin increases, resulting in insulin resistance. Thus, the insufficient secretion of insulin during pregnancy tends to lead to diabetes.

Risk factors for gestational diabetes include obesity, a history of diabetes in the family, and a history of poor blood glucose tolerance with a previous birth weight of more than 4000 g, previous fetal defects, a history of stillbirths or multiple miscarriages, maternal age (>35 years), and polycystic ovary syndrome. The diagnosis of gestational diabetes usually occurs around 24 to 28 weeks of gestation. The blood sugar level is drawn for screening one hour after drinking water containing 50 g of glucose. If the blood sugar value is greater than 140 mg/dl, a further 3-h oral glucose tolerance test is required to confirm the diagnosis. The 3-h oral glucose tolerance test requires fasting for 8 h and then testing the blood sugar level, followed by consuming a drink containing 100 g of glucose water, with further blood tests one, two, and three hours later. If two or more of the four blood glucose values exceed the standard value, it is defined as gestational diabetes.

The effects of diabetes on the fetus include: oversized baby, stillbirth, neonatal hypoglycemia, metabolic problems in the newborn, fetal defects, and others. An oversized baby refers to a baby whose birth weight exceeds 4000 g, and the probability of caesarean section and shoulder dystocia will increase due to increasing fetal weight. Since a large amount of sugar enters the fetus through the umbilical cord, the pancreas of the fetus will secrete a relatively large amount of insulin. Insulin itself is a good growth-stimulating hormone, which will cause the tissue of the fetus to proliferate and form an overweight baby.

Stillbirth can occur when maternal hyperglycemia persists, leading to placental vascular damage that reduces the supply of oxygen and nutrients to the infant. This reduction in oxygen can lead to physical injury or death of the baby, including stillbirth. This is less common in gestational diabetes and more common in pregestational diabetes, and as such pre-pregnancy diabetic mothers should pay close attention to the fetal condition at the end of pregnancy.

Neonatal hypoglycemia occurs when the baby’s pancreas secretes large amounts of insulin in response to the mother’s high blood sugar, but after the baby is born, the mother no longer supplies blood sugar. A lot of insulin can make the baby’s blood sugar too low (hypoglycemia; blood sugar < 40 mg/dL). At this time, the baby may be confused, emotionally tense, and even they have difficulty breathing or cramps.

Hyperglycemia and insulin imbalances often cause other metabolic problems and complications, such as jaundice and calcium or magnesium ion imbalances. The chance of a baby being born with diabetes is very low, especially if the mother has gestational diabetes. However, if the mother has type II diabetes before becoming pregnant, the risk of the baby having diabetes in adulthood will increase due to heredity. If the mother is type I diabetic, the child has a greater risk of having type I diabetes at birth.

Fetal defects occur in 2–3% of the general population. The risk of major defects for babies born to mothers with gestational diabetes is the same as that of the general population. But in the case of mothers with pregestational diabetes in the same group, the risk increases by about three to four times, especially if the pregnant woman has high blood sugar in the early pregnancy, as early pregnancy is an important period for the baby’s organ development and formation. The risk is directly related to blood sugar control. The most common fetal defects are in brain, spinal cord, and heart. Most fetal defects can be found in ultrasound examinations. In addition, the risk of chromosomal problems, such as Down syndrome, is not related to the presence or absence of diabetes. The key to reducing the risk of fetal defects is controlling blood sugar before pregnancy. Newborns born to diabetic mothers who may also have renal vein thrombosis, myocardial dysfunction, asymmetric hypertrophic cardiomyopathy, polycythemia, and left small colon syndrome, should be checked according to the clinical manifestations.

The effects of diabetes on the mother include: eye problems, kidney disease, high blood pressure, diabetic ketoacidosis, premature birth, infections, and cesarean delivery. Women with high blood pressure have a higher chance of developing gestational diabetes during pregnancy, and thus an increased chance of developing an overweight baby. The relative chance of a cesarean section also increases.

Mothers with pregestational diabetes who have poor blood sugar control may have significant vascular complications (especially eye and kidney problems). If diabetes has already caused damage to the small blood vessels in the eye, the damage can worsen during pregnancy, especially when blood sugar control is poor. This is extremely important. Women with diabetes are advised to see an ophthalmologist as soon as possible before pregnancy. Many retinal lesions are reversible and do not require treatment after pregnancy, but some may require close monitoring and laser therapy to avoid further damage during pregnancy. In rare cases, retinopathy worsens during pregnancy, such as active proliferative retinopathy. For such women, a cesarean delivery may be better than a vaginal delivery because the strain on the stomach can injure the tiny blood vessels in the eyes. The function of the kidney is to maintain and reabsorb good nutrients and excrete waste. If diabetes damages the kidney, it will cause loss of function, such as loss of urine protein. Having kidney disease greatly increases the risk of high blood pressure during pregnancy. Most kidney damage during pregnancy is a reversible change, but if the kidney is damaged too much, it may become irreversible and require dialysis. Therefore, monitoring of renal function should be carried out as early as possible, and renal function should be evaluated regularly during pregnancy if necessary.

High blood pressure caused by diabetes may worsen after pregnancy. Additionally, up to 50% of women with diabetes and high blood pressure may develop preeclampsia (high blood pressure, swelling (especially of the hands and face), protein in the urine).

Diabetic ketoacidosis only occurs in patients with type I diabetes, when the sugar in the blood is high and cannot be used by the cells. The body starts to use fat as energy, and the product of fat burning is called keto acid. If there is too much blood, a life-threatening situation called ketoacidosis may arise. Symptoms of ketoacidosis include high blood sugar, nausea, vomiting, abdominal pain, and keto acid in urine.

Premature birth can occur in a diabetes pregnancy that has been complicated by polyhydramnios, which may cause high blood sugar and cause the baby to urinate more frequently. Polyhydramnios may cause uterine contractions. In addition, infections, especially in the genitourinary tract (e.g., fungal infection), may also increase the risk of premature birth.

Cesarean sections are often used in the cases of overweight babies, or when the mother suffers complications that could lead to premature birth or high blood pressure [15].

A neural network is a computing system, including software and hardware, which uses a large number of simple connected artificial neurons to simulate the capabilities of biological neural networks. Artificial neurons are simple simulations of biological neurons that obtain information from the external environment or other artificial neurons, perform simple operations, and output the results to the external environment or other artificial neurons. An artificial neural network uses several microprocessors to represent neurons in the human brain, combines them into a neural network structure, and then selects an algorithm using mathematics and places it into the neural network. Training must be conducted in order to ensure the neural network works correctly, so that the neural network can learn repeatedly until each input properly corresponds to the required output. Therefore, before learning the neural network, a training pattern for the neural network must be established to have a reference in the learning process. The establishment of the training pattern comes from the input and output of the actual system or from previous experience. Generally, the indicators commonly used to summarize the information of the ROC curve are the area under the ROC curve and part of the area under the ROC curve. The area under the ROC curve is the global average sensitivity of the specificity, and part of the area under the ROC curve is the average sensitivity limited to the clinically meaningful range. It is an important task of diagnostic testing to compare the accuracy of new diagnostic tools with current standard diagnostic tools.

A confusion matrix, also known as an error matrix, is a standard format for expressing the evaluation of accuracy. It is represented by the matrix with n rows and n columns. Specific evaluation indexes are used for the overall accuracy, mapping accuracy, and user accuracy. These accuracy indexes reflect the accuracy of image classification from different aspects. In artificial intelligence, a confusion matrix is a visualization tool used for supervised learning, and it is generally called a matching matrix in unsupervised learning. For image accuracy evaluation, a confusion matrix is mainly used to compare the classification results with the actual measured values and then display the accuracy of the classification results in a confusion matrix. The confusion matrix is calculated by comparing the position and classification of each measured pixel with the corresponding position and classification in the classified image [16].

Hassan et al. [17] used 8 different features [18,19] and tested decision trees, k-NN, AdaBoost, Random Forest, Naive Bayes and XGBoost. The combination with the best results was AdaBoost and XGBoost. The area under the curve (AUC) score was 0.95.

## 3. Steps and Methods

### 3.1. Research Subjects

In this study, the outpatient examination data of a Taipei Municipal medical center was taken as the patient population and 15,000 women aged between 20 and 80 were selected as the samples. These women were patients who had gone to the hospital between 2018 and 2020 and between 2021 and 2022 and may or may not have been diagnosed with diabetes. The patients had eight characteristics that were considered for this study: number of pregnancies, plasma glucose level, diastolic blood pressure, sebum thickness, insulin level, BMI, diabetes pedigree function, and age.

### 3.2. Data Preprocessing

In this study, the collected data from tests on the patients in the past two years were used as predictors of the models. The data used in this study are explained below.

The input variables consisted of continuous data, including the number of pregnancies, plasma glucose level, diastolic blood pressure, sebum thickness, insulin level, BMI, diabetes pedigree function, and age.

**Figure 1:**
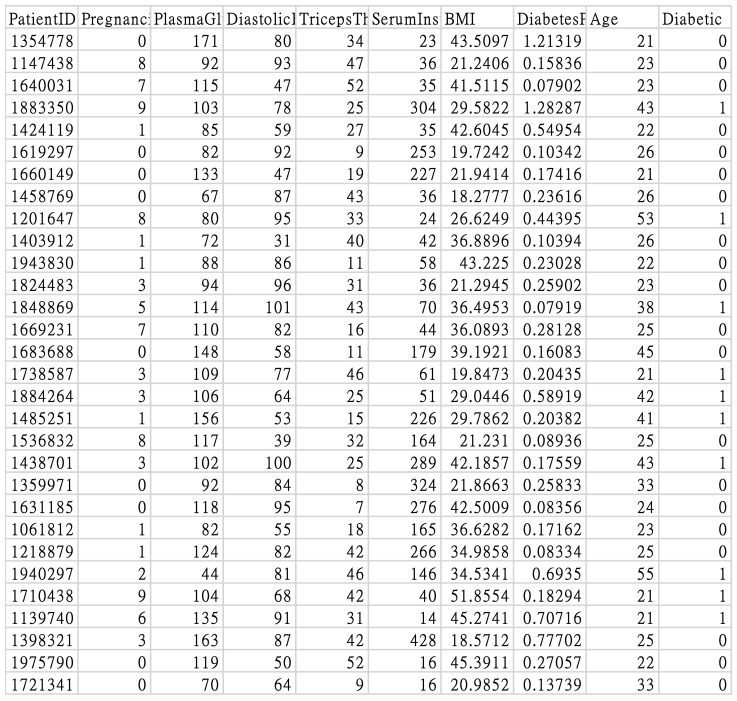
Original study data.

The output variables consisted of categorical data. The values indicated whether diabetes was diagnosed after two years, with 1 indicating diabetic and 0 indicating non-diabetic. The original data of this study (https://drive.google.com/file/d/1eAplOYO-k7ZYHj4uHAY1tEr8VTeaxS6u/view?usp=sharing, accessed on 21 September 2022) are shown in Figure 1.

### 3.3. Data Analysis and Classification

**Figure 2:**
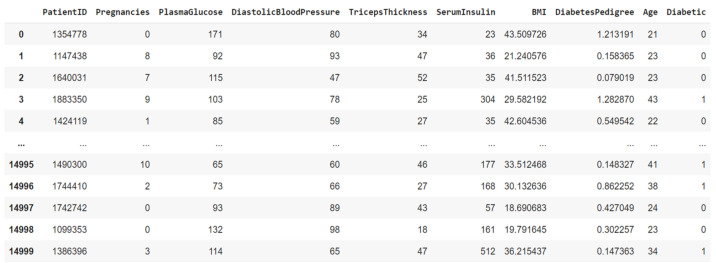
Data import for feature analysis.

**Figure 3:**
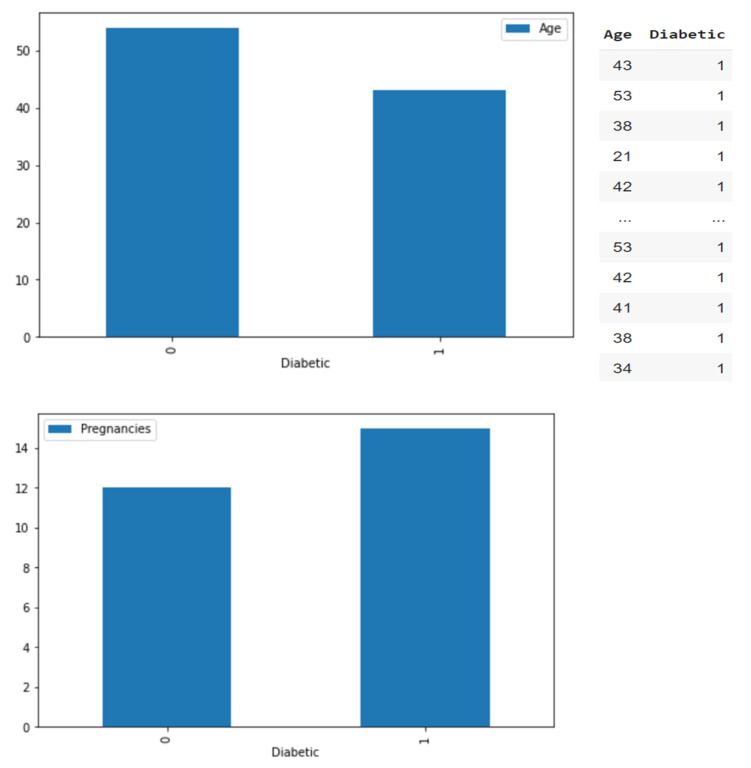
Diabetic/non-diabetic distribution.

**Figure 4:**
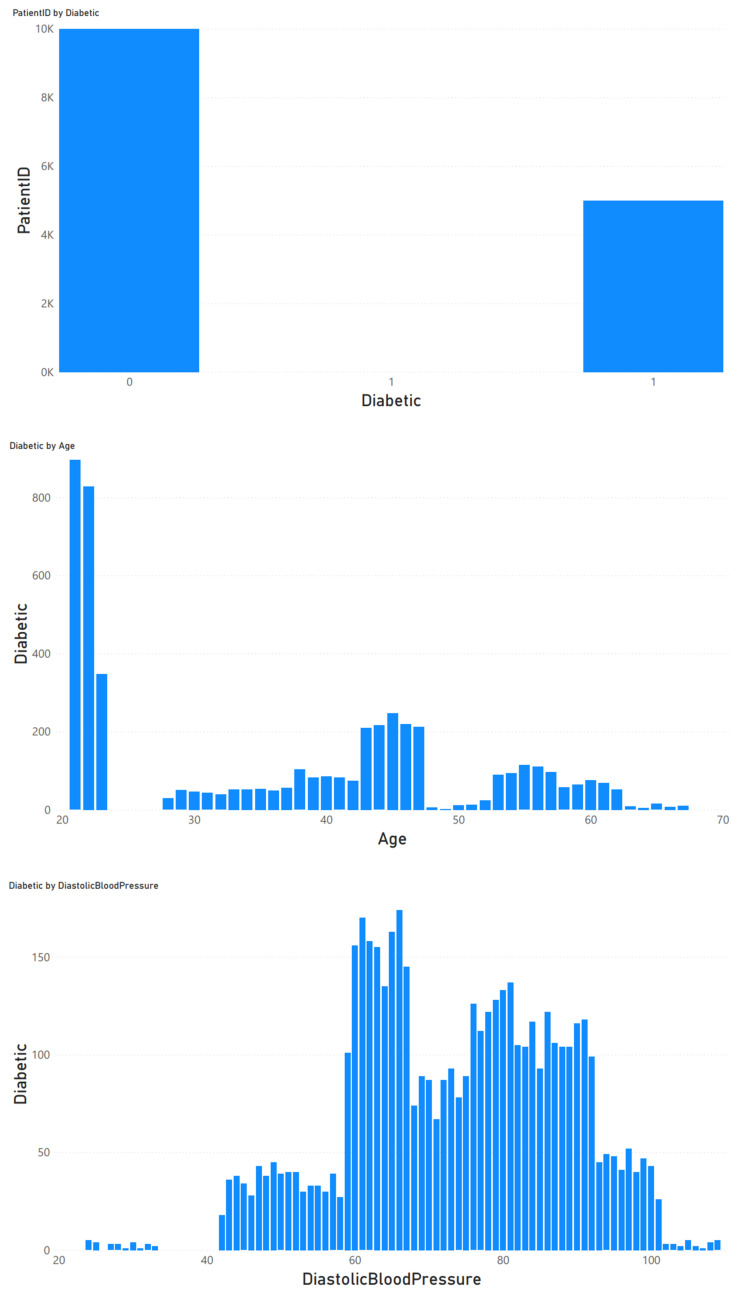

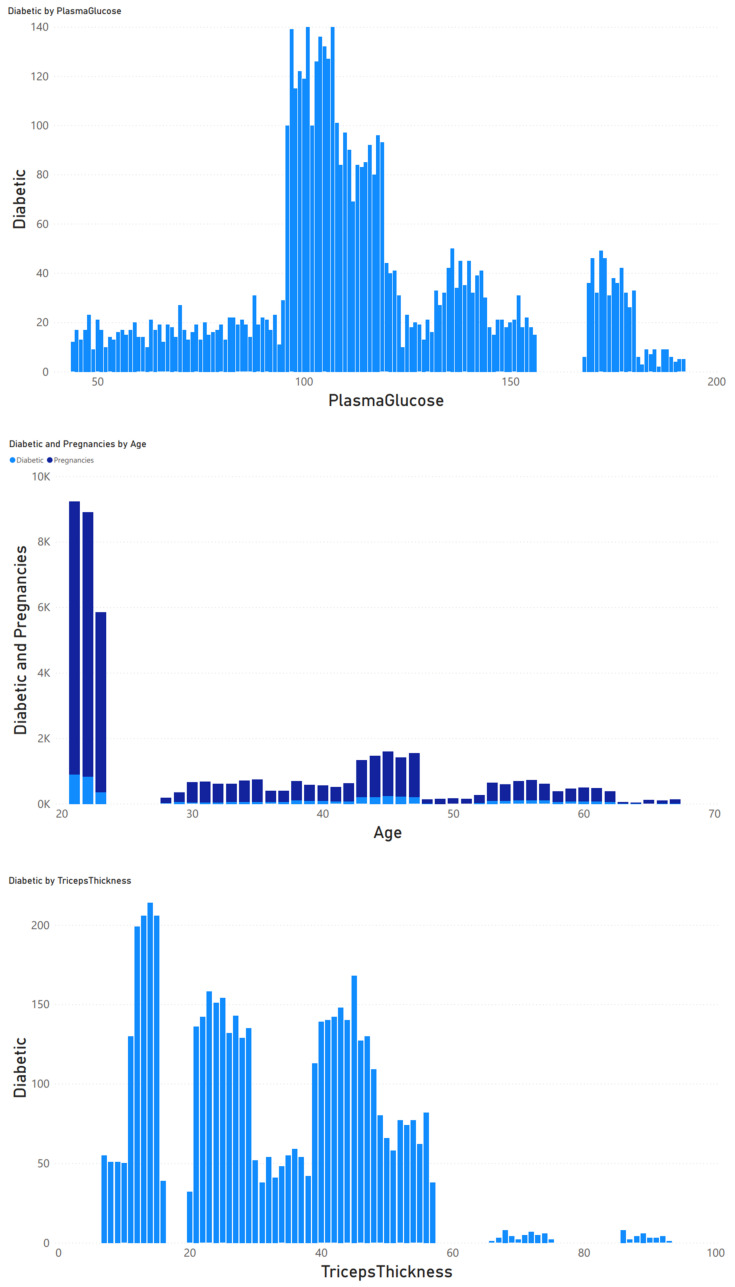
Correlation between diabetes and all variables.

The data were imported for feature analysis, as shown in Figure 2. Then, the distribution of diabetes was checked, as shown in Figure 3. Visualization tools were adopted to view the data distribution of each field when a patient was confirmed to have diabetes as well as the correlation between diabetes and all variables, as shown in Figure 4.

### 3.4. Model Evaluation Metrics

The following metrics were used to evaluate the proposed model [20,21,22].

When making predictions on events, there will be four types of results:

- True Positives (TP): someone with diabetes and was predicted to have diabetes.
- False Positives (FP): someone without diabetes was predicted to have diabetes.
- False Negatives (FN): someone with diabetes was not predicted to have diabetes.
- True Negatives (TN): someone without diabetes was not predicted to have diabetes.

Among the above four types, FP is also known as a Type I error, or α error. On the contrary, FN is also known as a Type II error, or β error. In terms of hypothesis testing, when H0 is false, H1 is predicted to be false.

Accuracy refers to the percentage of correct predictions made by the classifier when compared to the actual value of the label in the testing phase. It also represents the ratio of the number of correct assessments to the number of all assessments. The accuracy can be calculated using the following Equation (1) [23]:

(1) $$Accuracy=\frac{\left(TN+TP\right)}{\left(TN+TP+FN+FP\right)}$$

Precision is a significant measure for determining exactness. It states what percentage of instances the classifier labels as positive with respect to the total predictive positive instances, as shown in Equation (2):

(2) $$Precision=\frac{True\;Positive}{\left(True\;Positive+False\;Positive\right)}$$

Recall indicates what proportion of events that actually was of a certain class was classified by us as that class. It is the division of the true positives to all positives, as shown in Equation (3) [24]:

(3) $$Recall=\frac{True\;Positive}{\left(True\;Positive+False\;Negative\right)}$$

For classification problems that have a skewed distribution, accuracy by itself is not an appropriate metric. Instead, precision and recall are much more representative.

These two metrics of precision and recall can be combined to get the F1 score, which is the weighted average (harmonic mean) of the precision and recall scores. The score ranges from 0 to 1, with 1 being the best possible F1 score (the harmonic mean is employed when dealing with ratios), as shown in Equation (4):

(4) $$F1=\frac{2}{\left(\frac{1}{Precision}\right)+\left(\frac{1}{Recall}\right)}$$

The receiver operator characteristic (ROC) compares the change between the true positive rate (TPR) and the false positive rate (FPR) under various decision thresholds.

The size of the area under the curve (AUC) can be regarded as the performance of the model and is often used to compare the performance of multiple models.

Therefore, the TPR and FPR can be calculated under various thresholds as sample points.

If AUC = 1, it means that the model is perfect.

If AUC > 0.5, it means that the classification effects of the model is better than random guessing, and the model has predictive value.

If AUC = 0.5, it means that the classification effects of the model is the same as random guessing, and the model has no predictive value.

If AUC < 0.5, it means that the classification effects of the model is worse than random guessing. However, if reverse prediction is performed, it will be better than random guessing.

**Figure 5:**
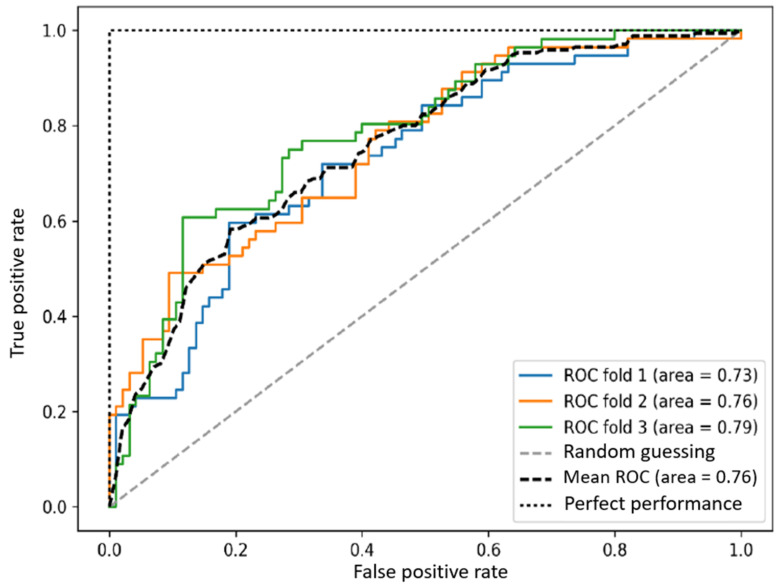
ROC curve.

When all sample points are connected to form a line, this is called the ROC curve. The closer this line is to the top, the higher the TPR, that is, the higher the ratio of correct judgment. In other words, the larger the area covered under the ROC curve (AUC), the better the performance [25], as shown in Figure 5.

### 3.5. Machine Learning Model

**Figure 6:**
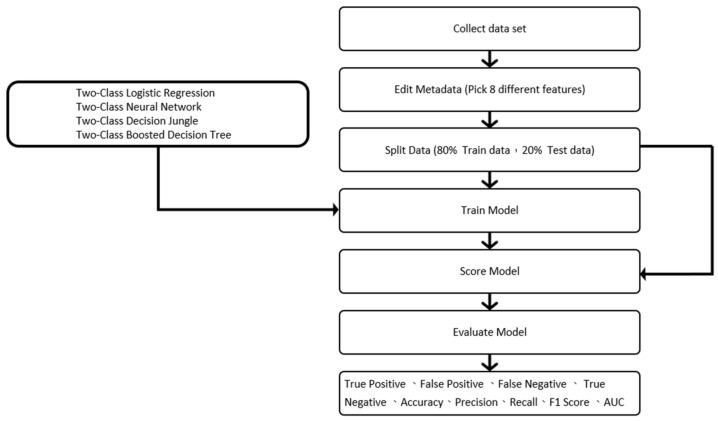
The study flow chart.

**Figure 7:**
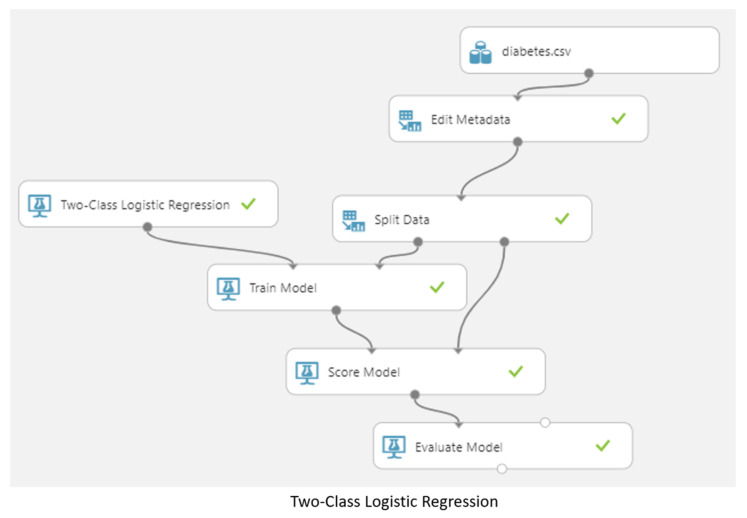

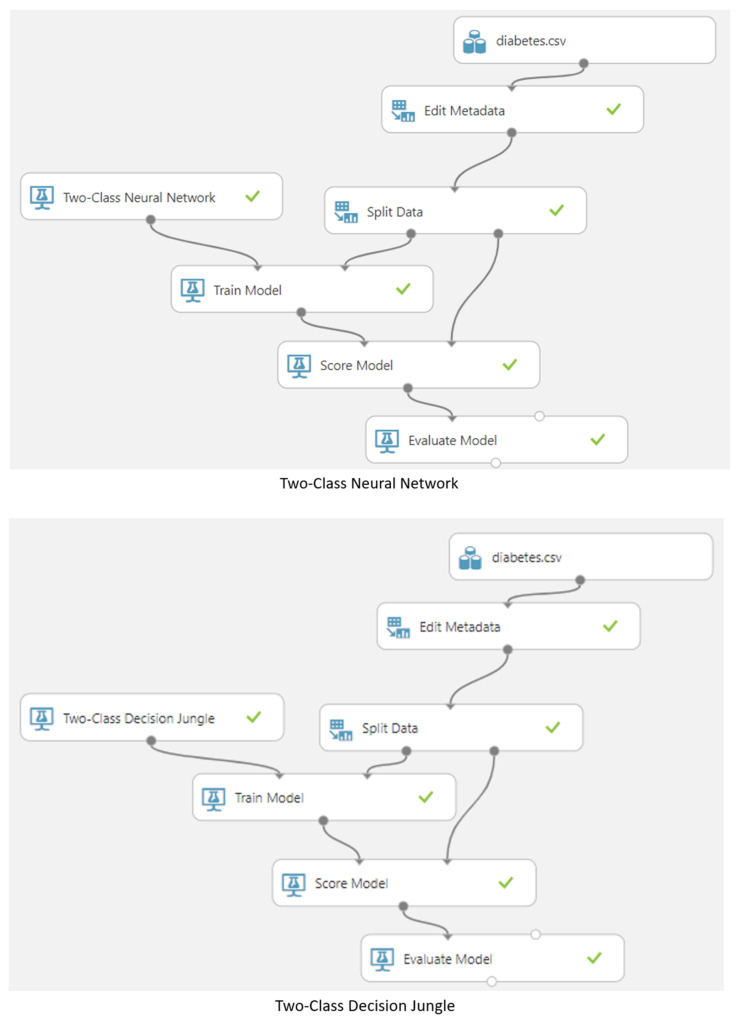

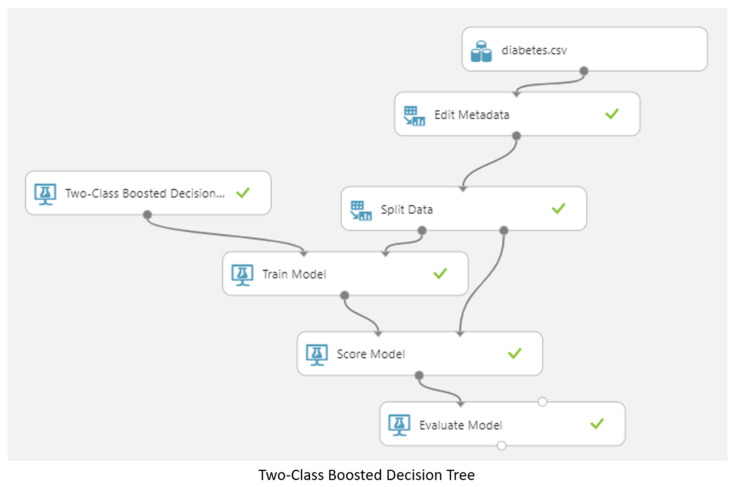
Predictions made by the four different models.

**Figure 8:**
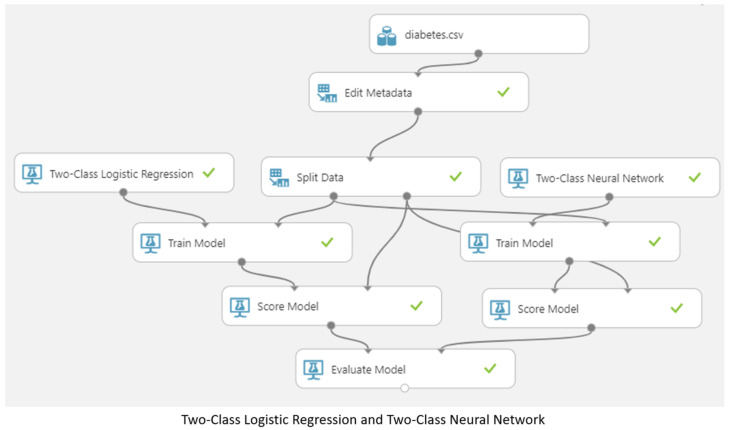

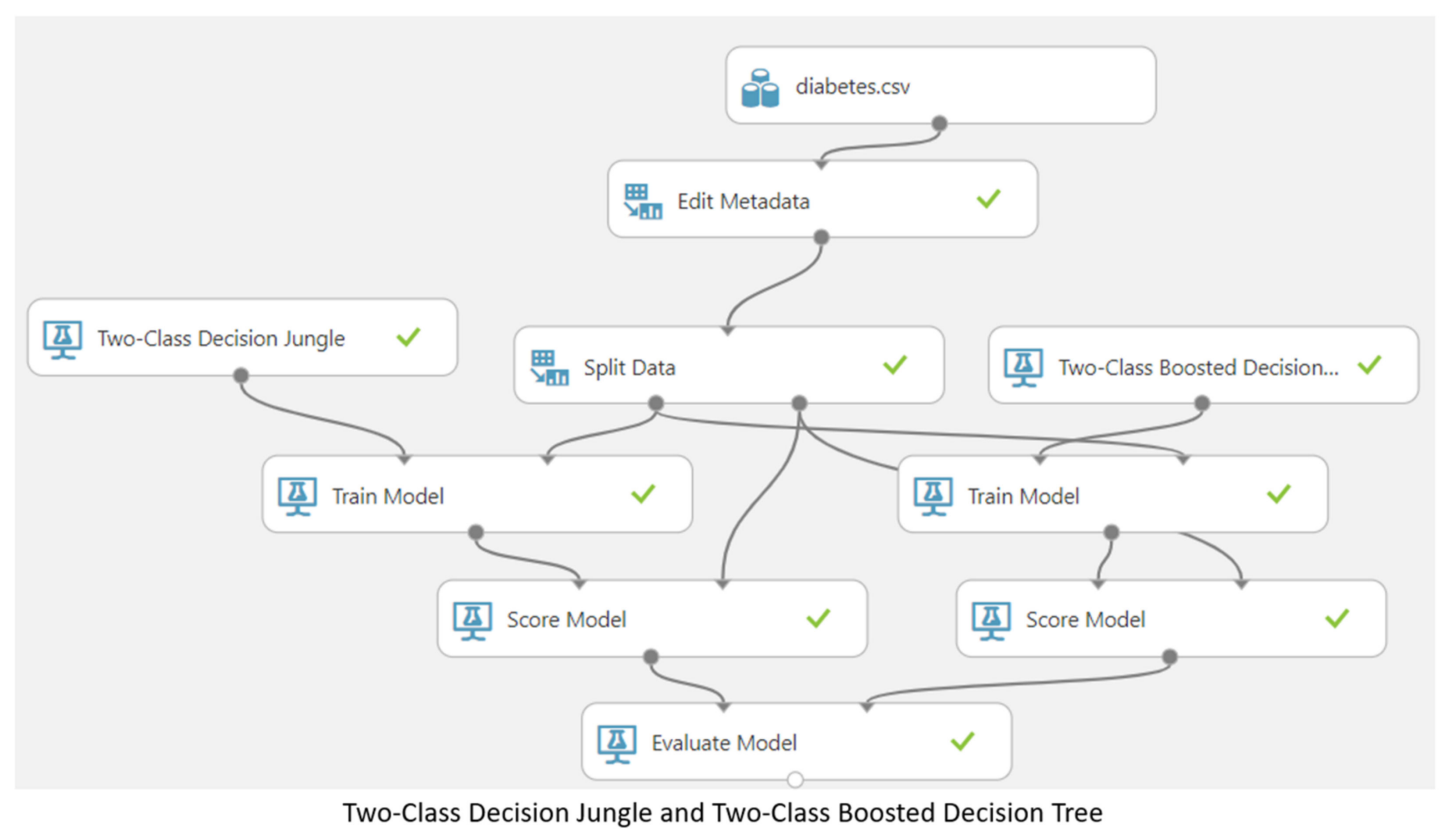
Cross-validation and comparison of the four different models.

In this study flow chart, as shown in Figure 6, 150,000 pieces of data were divided into training data and test data, of which 80% were used as training data and 20% as test data [26]. Four different models (two-class logistic regression, two-class neural network, two-class decision jungle, and two-class boosted decision tree) were used to make predictions [27,28,29,30], as shown in Figure 7, after which cross-validation and comparisons were made [31,32], as shown in Figure 8. Finally, the true positive, false positive, false negative, true negative, accuracy, precision, recall, F1 score, and AUC results were obtained [33,34].

## 4. Results and Discussion

**Figure 9:**
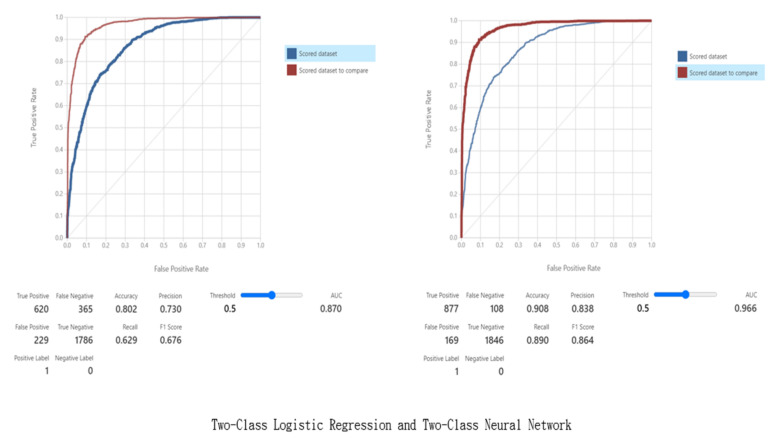

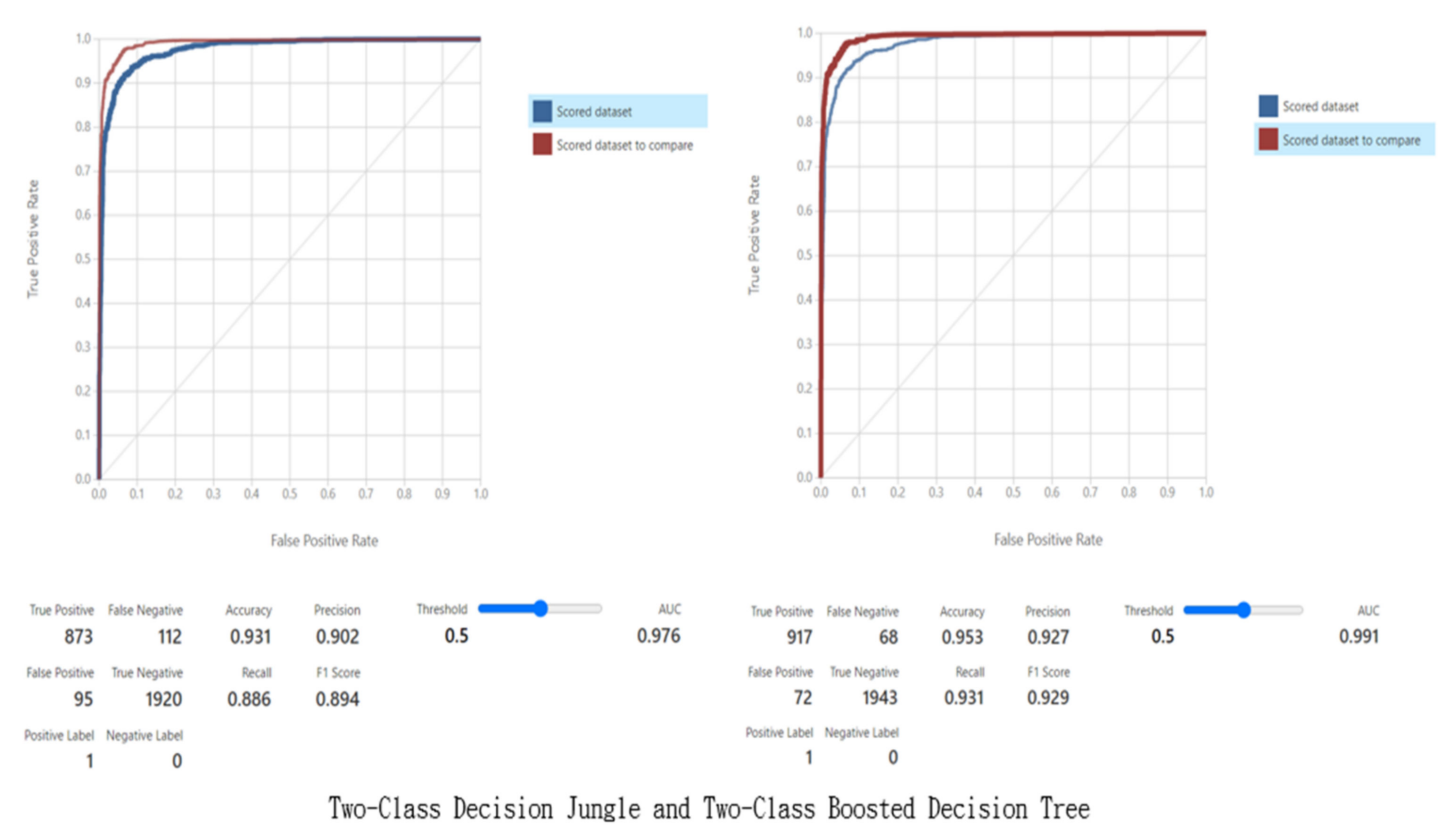
Verification results.

Through the above data input and feature classification, this study showed that the subjects were prone to developing diabetes (especially during pregnancy) due to low insulin absorption, high cholesterol levels, or elevated blood pressure [35,36]. After model training, storing of the result models, and model testing were completed, cross-validation and comparison were carried out. The verification results of the metrics used for evaluation of the model, including the true positive, false positive, false negative, true negative, accuracy, precision, recall, F1 score, and AUC values, were obtained in this study, as shown in Figure 9. A summary of the verification results is shown in Table 1.

To verify the values shown in Table 1, and to thus check the validity of the models, the values were substituted into the aforementioned Formulas (1)–(4) to obtain the following values, which show that the verification is correct.

$$Accuracy=\frac{\left(TN+TP\right)}{\left(TN+TP+FN+FP\right)}=\frac{\left(1786+620\right)}{\left(1786+620+365+229\right)}=0.802$$

$$Precision=\frac{True\;Positive}{\left(True\;Positive+False\;Positive\right)}=\frac{620}{\left(620+229\right)}=0.730$$

$$Recall=\frac{True\;Positive}{\left(True\;Positive+False\;Negative\right)}=\frac{620}{\left(620+365\right)}=0.629$$

$$F1=\frac{2}{\left(\frac{1}{\mathrm{Precision}}\right)+\left(\frac{1}{\mathrm{Recall}}\right)}=\frac{2}{\left(\frac{1}{0.730}\right)+\left(\frac{1}{0.629}\right)}=0.676$$

## 5. Conclusions

Diabetes is one of the most serious chronic diseases today, and early diagnosis can greatly improve patients’ chances of managing it. The latest developments in machine intelligence can be used to improve the understanding of the factors that lead to the onset of diabetes. This study used eight different characteristics (number of pregnancies, plasma glucose level, diastolic blood pressure, sebum thickness, insulin level, BMI, diabetes pedigree function, and age) for data preprocessing. After training, testing, cross-validation, and comparison, this study obtained the data for the model performance analysis.

The results showed that all models achieved good results; however, the best models were the two-class decision jungle and two-class boosted decision tree. The area under the curve (AUC) was selected as the performance indicator and AUC scores of 0.976 and 0.991 were achieved, which was better than expected based on the literature Hasan et al [27].

These results provided an improvement to the existing prediction methods for diabetes. It is worthwhile to explore these models using unsupervised machine learning and deep learning techniques in future research [37].

## Figures and Tables

**Table 1:** Verification results.

Metrics for Evaluation of the Model									
Model	True Positive	False Positive	False Negative	True Negative	Accuracy	Precision	Recall	F1 Score	AUC
Two-Class Logistic Regression	620	229	365	1786	0.802	0.73	0.629	0.676	0.87
Two-Class Neural Network	877	169	108	1846	0.908	0.838	0.89	0.864	0.966
Two-Class Decision Jungle	873	95	112	1920	0.931	0.902	0.886	0.894	0.976
Two-Class Boosted Decision Tree	917	72	68	1943	0.953	0.927	0.931	0.929	0.991

## Data Availability

The research data related to this work are included within the manuscript. For more information on the data, contact the corresponding authors.

## References

[1] (2021). International Diabetes Federation. https://diabetesatlas.org/atlas/tenth-edition/.

[2] American Diabetes Association (2009). Standards of medical care in diabetes—2009. Diabetes Care.

[3] Stephen C., Daniel D. (2009). The value of early detection of type 2 diabetes. Curr. Opin. Endocrinol. Diabetes Obes..

[4] Dagliati A., Marini S., Sacchi L., Cogni G., Teliti M., Tibollo V., De Cata P., Chiovato L., Bellazzi R. (2018). Machine Learning Methods to Predict Diabetes Complications. J. Diabetes Sci. Technol..

[5] Tapp R.J., Shaw J.E., Zimmet P.Z., Balkau B., Chadban S.J., Tonkin A.M., Welborn T.A., Atkins R.C. (2004). Albuminuria is evident in the early stages of diabetes onset: Results from the Australian Diabetes, Obesity, and Lifestyle Study (AusDiab). Am. J. Kidney Dis..

[6] Katarya R., Maan S. Stress Detection using Smartwatches with Machine Learning: A Survey. Proceedings of the 2020 International Conference on Electronics and Sustainable Communication Systems (ICESC).

[7] Expert Committee on the Diagnosis and Clasification of Diabetes Mellitus (2002). American Diabetes Association: Clinical practice recommendations 2002. Diabetes Care..

[8] Joshi R.D., Dhakal C.K. (2021). Predicting Type 2 Diabetes Using Logistic Regression and Machine Learning Approaches. Int. J. Environ. Res. Public Health.

[9] Kavakiotis I., Tsave O., Salifoglou A., Maglaveras N., Vlahavas I., Chouvarda I. (2017). Machine Learning and Data Mining Methods in Diabetes Research. Comput. Struct. Biotechnol. J..

[10] Rodríguez-Rodríguez I., Chatzigiannakis I., Rodríguez J.-V., Maranghi M., Gentili M., Zamora-Izquierdo M.-Á. (2019). Utility of Big Data in Predicting Short-Term Blood Glucose Levels in Type 1 Diabetes Mellitus Through Machine Learning Techniques. Sensors.

[11] Kopitar L., Kocbek P., Cilar L., Sheikh A., Stiglic G. (2020). Early detection of type 2 diabetes mellitus using machine learning-based prediction models. Sci. Rep..

[12] Makroum M.A., Adda M., Bouzouane A., Ibrahim H. (2022). Machine Learning and Smart Devices for Diabetes Management: Systematic Review. Sensors.

[13] Ahmad H.F., Mukhtar H., Alaqail H., Seliaman M., Alhumam A. (2021). Investigating Health-Related Features and Their Impact on the Prediction of Diabetes Using Machine Learning. Appl. Sci..

[14] Jian Y., Pasquier M., Sagahyroon A., Aloul F. (2021). A Machine Learning Approach to Predicting Diabetes Complications. Healthcare.

[15] Jagannathan R., Neves J.S., Dorcely B., Chung S.T., Tamura K., Rhee M., Bergman M. (2020). The Oral Glucose Tolerance Test: 100 Years Later. Diabetes Metab. Syndr. Obes..

[16] Markoulidakis I., Rallis I., Georgoulas I., Kopsiaftis G., Doulamis A., Doulamis N. (2021). Multiclass Confusion Matrix Reduction Method and Its Application on Net Promoter Score Classification Problem. Technologies.

[17] Larabi-Marie-Sainte S., Aburahmah L., Almohaini R., Saba T. (2019). Current Techniques for Diabetes Prediction: Review and Case Study. Appl. Sci..

[18] Meng X.-H., Huang Y.-X., Rao D.-P., Zhang Q., Liu Q. (2013). Comparison of three data mining models for predicting diabetes or prediabetes by risk factors. Kaohsiung J. Med. Sci..

[19] Abdulhadi N., Al-Mousa A. Diabetes Detection Using Machine Learning Classification Methods. Proceedings of the 2021 International Conference on Information Technology (ICIT).

[20] Mujumdar A., Vaidehi V. (2019). Diabetes Prediction using Machine Learning Algorithms. Procedia Comput. Sci..

[21] Birjais R., Mourya A.K., Chauhan R., Kaur H. (2019). Prediction and diagnosis of future diabetes risk: A machine learning approach. SN Appl. Sci..

[22] Katarya R., Srinivas P. (2020). Identifying Risks in Cardiovascular Disease Using Supervised Machine Learning Algorithms. ICICNIS 2020. https://ssrn.com/abstract=3769903.

[23] Gadekallu T.R., Khare N., Bhattacharya S., Singh S., Maddikunta P.K.R., Ra I.-H., Alazab M. (2020). Early Detection of Diabetic Retinopathy Using PCA-Firefly Based Deep Learning Model. Electronics.

[24] Nadeem M.W., Goh H.G., Ponnusamy V., Andonovic I., Khan M.A., Hussain M. (2021). A Fusion-Based Machine Learning Approach for the Prediction of the Onset of Diabetes. Healthcare.

[25] Ryu K.S., Lee S.W., Batbaatar E., Lee J.W., Choi K.S., Cha H.S. (2020). A Deep Learning Model for Estimation of Patients with Undiagnosed Diabetes. Appl. Sci..

[26] Rahul, Katarya R. A Review: Predicting the Performance of Students Using Machine learning Classification Techniques. Proceedings of the 2019 Third International Conference on I-SMAC (IoT in Social, Mobile, Analytics and Cloud) (I-SMAC).

[27] Hasan M.K., Alam M.A., Das D., Hossain E., Hasan M. (2020). Diabetes Prediction Using Ensembling of Different Machine Learning Classifiers. IEEE Access.

[28] Ghosh P., Azam S., Karim A., Hassan M., Roy K., Jonkman M. (2021). A Comparative Study of Different Machine Learning Tools in Detecting Diabetes. Procedia Comput. Sci..

[29] Lai H., Huang H., Keshavjee K., Guergachi A., Gao X. (2019). Predictive models for diabetes mellitus using machine learning techniques. BMC Endocr. Disord..

[30] Katarya R., Jain S. Comparison of Different Machine Learning Models for Diabetes Detection. Proceedings of the 2020 IEEE International Conference on Advances and Developments in Electrical and Electronics Engineering (ICADEE).

[31] Katarya R., Srinivas P. Predicting Heart Disease at Early Stages Using Machine Learning: A Survey. Proceedings of the 2020 International Conference on Electronics and Sustainable Communication Systems (ICESC).

[32] Deberneh H.M., Kim I. (2021). Prediction of Type 2 Diabetes Based on Machine Learning Algorithm. Int. J. Environ. Res. Public Health.

[33] Sisodia D., Sisodia D.S. (2018). Prediction of diabetes using classification algorithms. Procedia Comput. Sci..

[34] Kaur H., Kumari V. (2022). Predictive modelling and analytics for diabetes using a machine learning approach. Appl. Comput. Inform..

[35] Battineni G., Sagaro G.G., Nalini C., Amenta F., Tayebati S.K. (2019). Comparative Machine-Learning Approach: A Follow-Up Study on Type 2 Diabetes Predictions by Cross-Validation Methods. Machines.

[36] Forouhi N.G., Wareham N.J. (2010). Epidemiology of diabetes. Medicine.

[37] Gupta A., Katarya R. (2020). Social media based surveillance systems for healthcare using machine learning: A systematic review. J. Biomed. Inform..

